# Heterotaxy Syndrome Diagnosed in an Adult

**DOI:** 10.31486/toj.24.0021

**Published:** 2024

**Authors:** Gizem Guney, Furkan Aydinli, Selime Aksit, Kenan Kadirli, Musa Salmanoglu

**Affiliations:** ^1^ Department of Internal Medicine, Sultan 2. Abdul Hamid Khan Educational and Research Hospital, Istanbul, Turkey; ^2^ Department of Radiology, Sultan 2. Abdul Hamid Khan Educational and Research Hospital, Istanbul, Turkey

**Keywords:** *Annular pancreas*, *cardiovascular abnormalities*, *heterotaxy syndrome*, *levocardia*

## Abstract

**Background:** Heterotaxy syndrome, a condition in which the internal organs are abnormally arranged in the thorax or abdomen, is generally diagnosed early in life, often during the neonatal period.

**Case Report:** We present the case of a 42-year-old male who was incidentally diagnosed with polysplenia syndrome and subsequently diagnosed with heterotaxy syndrome. Upon further investigation, he was determined to have a sinus venosus type atrial septal defect. Furthermore, the patient's inferior vena cava was interrupted in the infrarenal region and continued as the azygos vein with a coincidental retroaortic left renal vein, an anatomic variation unrelated to heterotaxy syndrome. Because of his minimal cardiac anomalies, the patient remained undiagnosed until adulthood.

**Conclusion:** According to our research, this case is the first report of a patient with heterotaxy syndrome and a sinus venosus type atrial septal defect. This case augments the available information about the variations of this rare syndrome.

## INTRODUCTION

Heterotaxy syndrome is a rare abnormality—0.81 per 10,000 births—in which the right-left axis of the thoracoabdominal organs is reversed during embryologic development.^1^ Many anatomic variations of the organs are seen in patients with heterotaxy syndrome, principally cardiovascular malformations, intestinal malformations, asplenia/polysplenia, and inferior vena cava anomalies. Situs solitus is the medical term used to describe organs in their normal arrangement in the thorax and abdomen. Organ displacement in the mirror image of the normal is called situs inversus totalis. Situs ambiguus is the term used for variations of organ and vasculature localization. The terms situs ambiguus and heterotaxy syndrome are used interchangeably. Because every case of situs ambiguus is unique, patients must be evaluated individually.^2^ Polysplenia syndrome is a congenital condition characterized by multiple spleens and is a type of heterotaxy syndrome. Anatomically, polysplenia syndrome is characterized by multiple splenules without a larger parent spleen, occurring either on one side of the body or dispersed on both sides. Polysplenia syndrome is associated with congenital heart disease and with gastrointestinal, genitourinary, and vascular abnormalities.

Because heterotaxy is a rare disorder, evidence-based data for diagnosis, treatment, and follow-up are insufficient. We present the case of a 42-year-old male with an incidental finding of heterotaxy polysplenia syndrome in which the inferior vena cava was continuous with the azygous vein.

## CASE REPORT

A 42-year-old male presented to the internal medicine outpatient clinic to obtain a second opinion on his liver enzyme levels. Moderate liver enzyme elevation and hyperlipidemia had been incidentally detected in the patient 3 years prior. During the preceding 3 years, possible causes of liver function abnormality such as hepatitis viruses, Epstein-Barr virus, other infectious agents, autoimmune disorders, metabolic accumulation disorders, and malignancies were ruled out. The patient's medical history included 2 cardiovascular surgeries. When the patient was 7 years old, a sinus venosus type atrial septal defect was corrected. At age 31 years, the patient underwent aortic valve replacement and aortic tube graft placement for aortic regurgitation due to bicuspid aortic valve and ascending aorta aneurysm. The patient's medications were warfarin and atorvastatin only, and he had no additional comorbidities.

At our clinic, laboratory workup showed alanine aminotransferase of 127 U/L (reference range, 4-36 U/L), aspartate aminotransferase of 109 U/L (reference range, 8-33 U/L), alkaline phosphatase of 25 U/L (reference range, 44-147 U/L), and gamma-glutamyl transferase of 38 U/L (reference range, 0-30 U/L). The patient's physical examination was normal.

**Figure 1.**
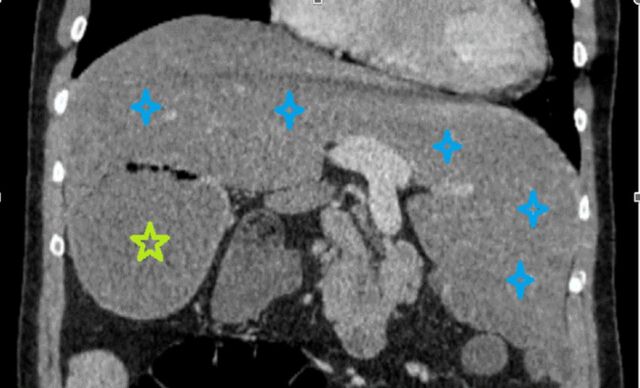
Portal venous phase coronal computed tomography through the hilum of the centrally localized liver and portal vein shows the liver filling the right and predominantly left upper quadrants of the abdomen (right and left liver lobes marked with blue stars). The corpus section of the stomach (green star) is located in the right upper quadrant.

An abdominal ultrasound, taken 3 months prior to presentation at our clinic, showed hepatomegaly with a length of 170 mm, as well as a 50-mm mass-like structure of indistinguishable etiology in the lower right region of the liver. At our clinic, contrast-enhanced abdominal computed tomography (CT) showed that the mass-like structure on the right was the stomach, and the liver was completely occupying the right and left upper quadrants of the abdomen, indicating a heterotaxy anomaly (Figure 1). Intrahepatic bile ducts, vena porta branches, and the gallbladder were normal, while the stomach was located on the right instead of the normal left-sided anatomic position.

**Figure 2.**
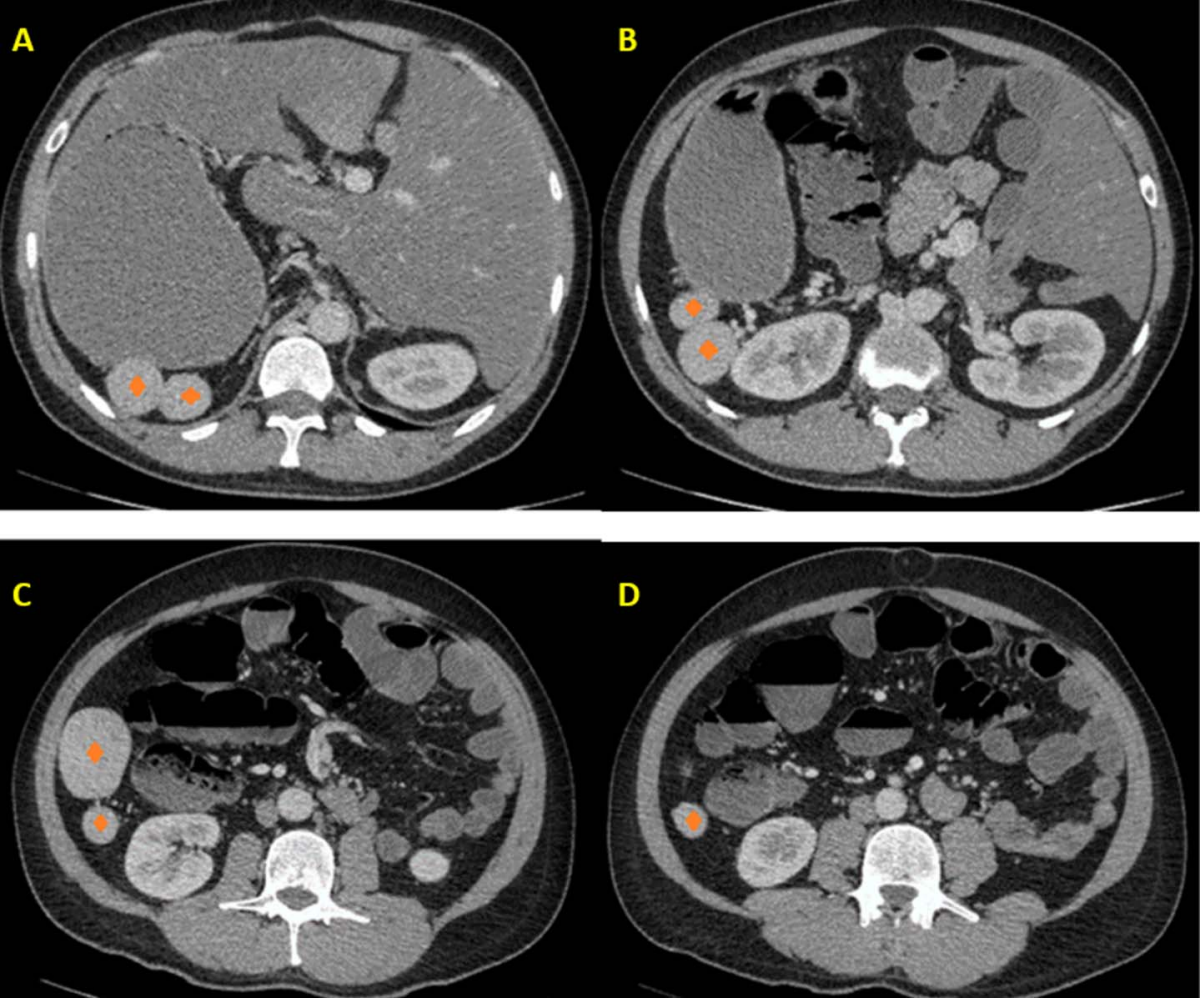
(A-D) Axial computed tomography craniocaudal sequential sections show multiple, well-circumscribed, soft tissue density nodular splenules (orange diamonds) located to the right of the abdomen with the ipsilaterally located stomach.

CT also revealed polysplenia. A total of 8 spleens—the largest of which was 56 × 45 mm—were observed in the right upper quadrant of the abdomen (Figure 2). Functional hyposplenism was not considered because the patient did not have a history of frequent infections.

**Figure 3.**
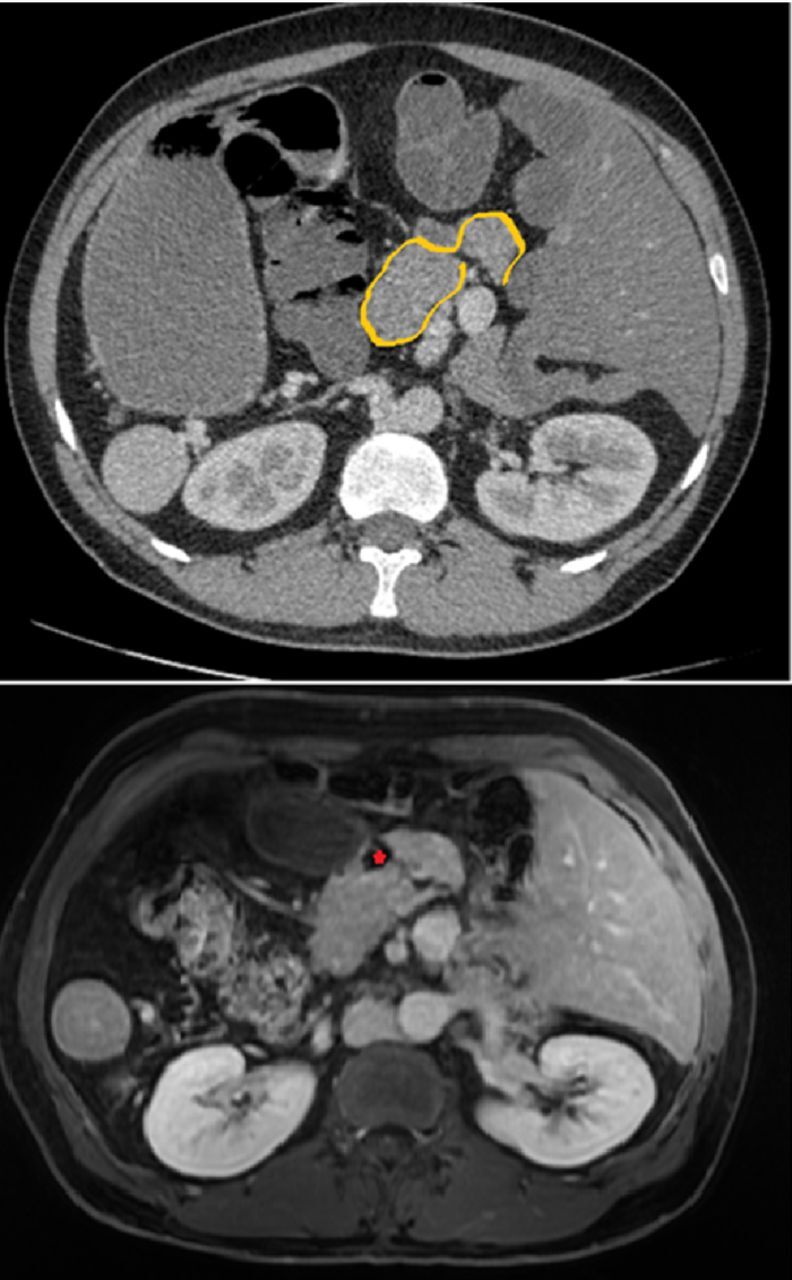
(Top) Fat-saturated axial T1-weighted computed tomography image shows the partial annular pancreas (pancreatic tissue outlined in yellow) with the duodenum located anteriorly. (Bottom) Contrast-enhanced axial fat-saturated computed tomography image shows the pancreatic tissue almost completely surrounding the second section of the duodenum (red star).

The pancreas almost completely surrounded the second part of the duodenum and was evaluated as annular pancreas with normal function (Figure 3). Annular pancreas is a rare congenital anomaly characterized by partial or complete circumferential encasement of the second part of the duodenum by a band of pancreatic tissue.^3^

**Figure 4.**
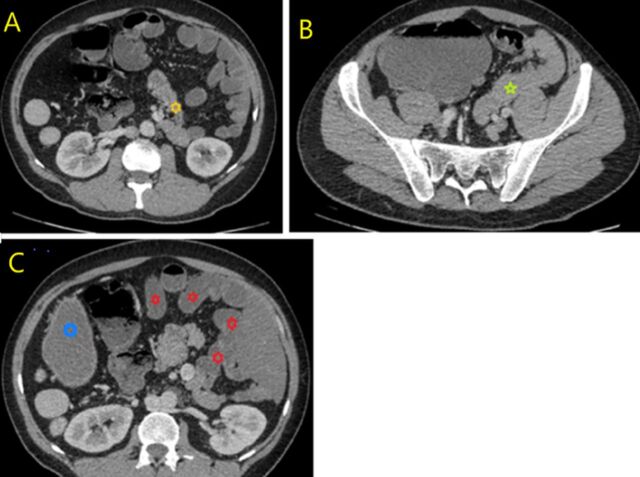
Intestinal malrotation without volvulus. Contrast-enhanced axial fat-saturated computed tomography image shows (A) abnormal position of the duodenojejunal junction (yellow asterisk), (B) jejunal loops located predominantly in the left lower quadrant (green star), and (C) ileal loops located predominantly in the left upper quadrant (red asterisks) with the stomach in the right quadrant (blue circle).

The gastrointestinal system showed subclinical ileus and intestinal malrotation (Figure 4). The duodenojejunal junction was located posteroinferior to the duodenal bulb and to the left of the midline. Jejunal loops were located in the lower left quadrant, while the ileal loops were located in the upper left quadrant. The cecum was in the normal position but was dilated. The patient had no historic or active complaints about the gastrointestinal system, and his physical examination findings were normal.

**Figure 5.**
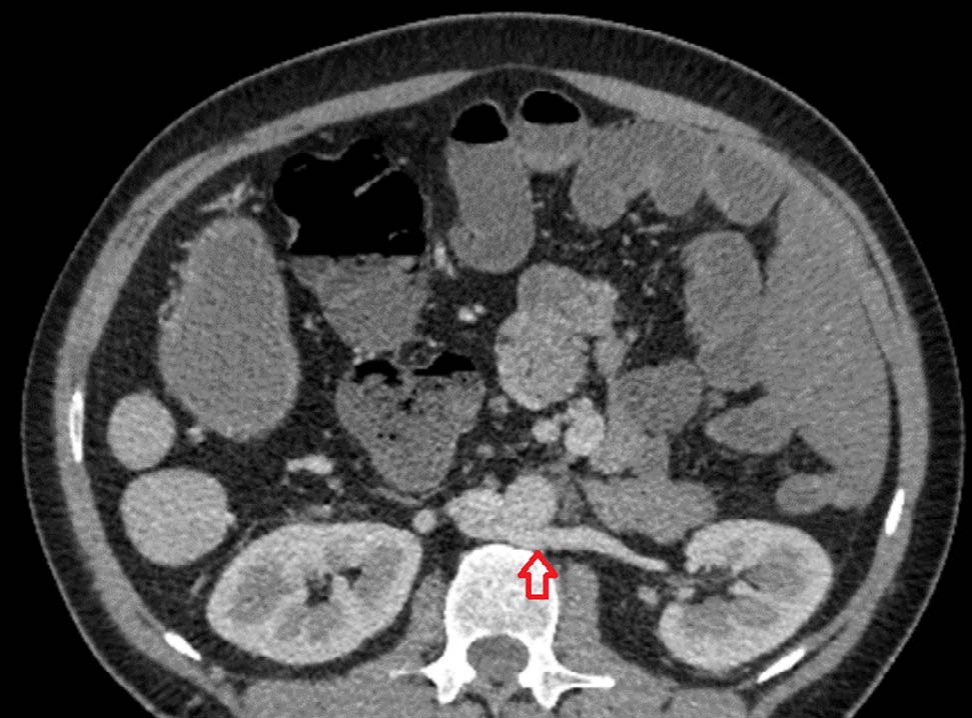
Contrast-enhanced axial fat-saturated computed tomography image shows the retroaortic course of the left renal vein (red arrow).

**Figure 6.**
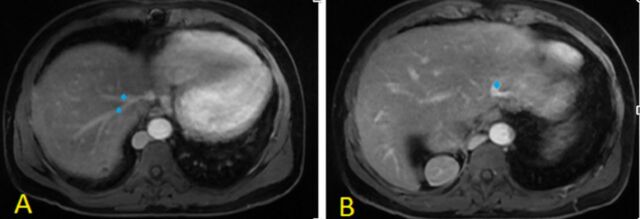
(A, B) Postcontrast axial magnetic resonance imaging shows the hepatic veins (blue diamonds), draining directly into the right atrium.

**Figure 7.**
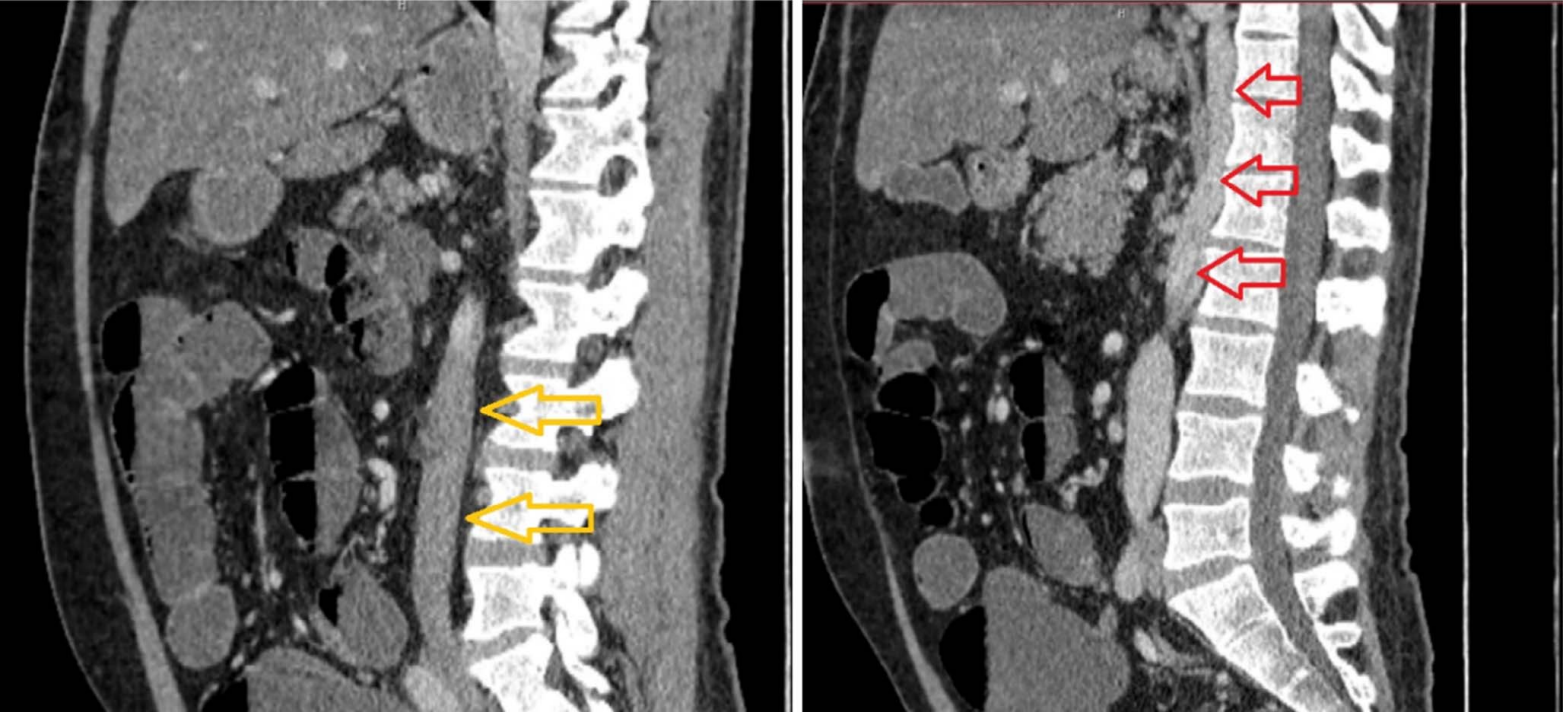
Consecutive sagittal plane computed tomography images show (left image) the interruption of the vena cava (yellow arrows) at the suprarenal level and (right image) continuity with the dilated azygos vein (red arrows).

**Figure 8.**
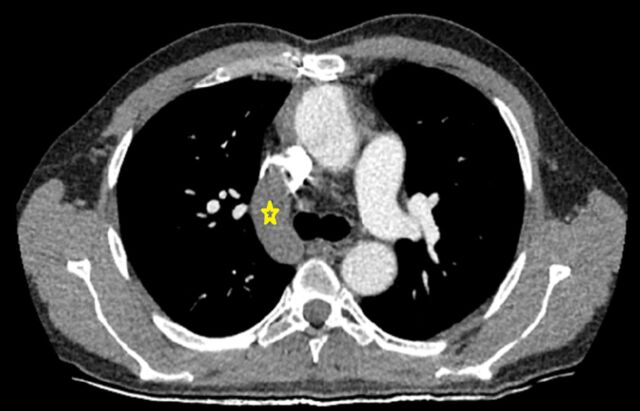
Fat-saturated contrast-enhanced computed tomography through the main pulmonary artery shows the junction of the dilated azygos vein (yellow star) and the superior vena cava.

The position and size of both kidneys were normal. However, the patient's left renal vein was retroaortic (ie, located behind the aorta, in front of the vertebrae) and drained into the inferior vena cava (Figure 5). Retroaortic left renal vein is an anatomic variant with a reported incidence of 3.6% in a study of 1,856 patients.^4^ The inferior vena cava was in the normal position to the right of the abdominal aorta, but the liver segment of the inferior vena cava was absent. Hepatic veins drained directly into the right atrium (Figure 6). The inferior vena cava continued as the enlarged azygos vein (Figures 7 and 8).

**Figure 9.**
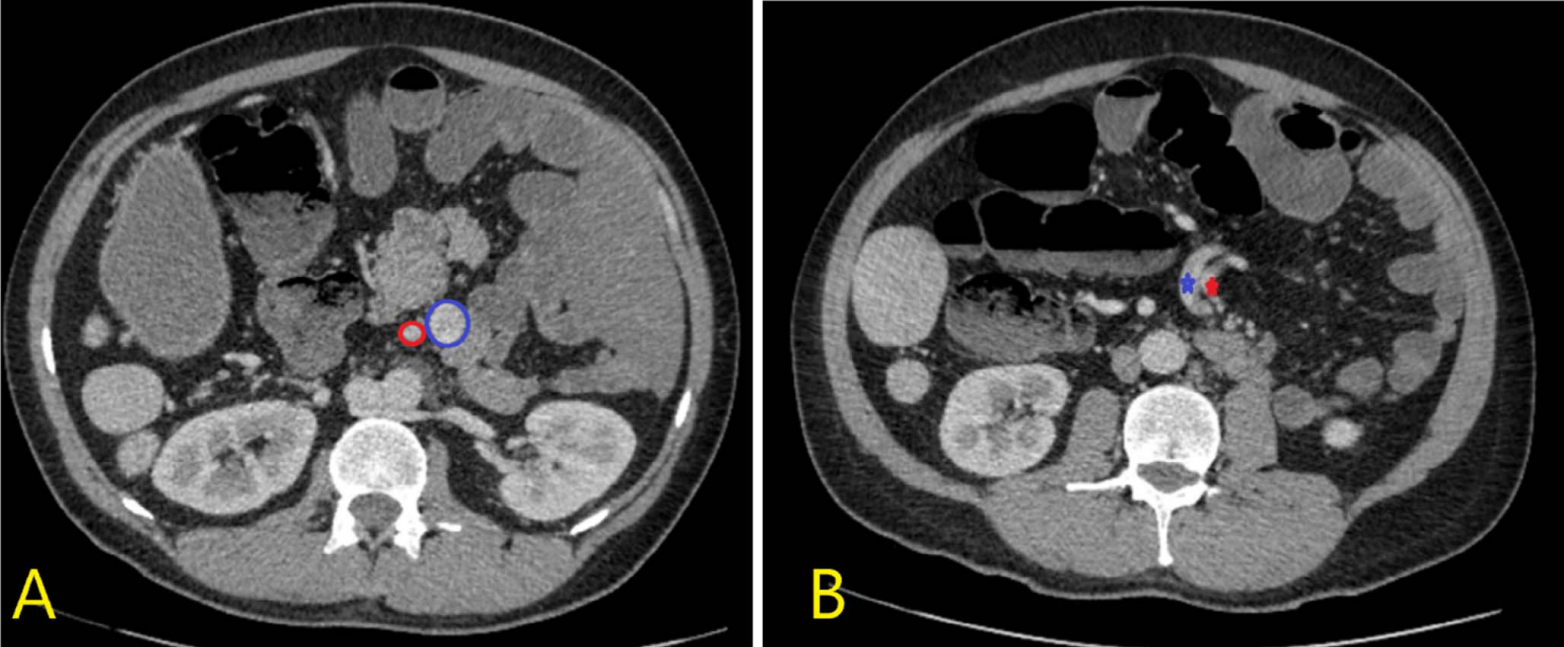
(A) Fat-saturated contrast-enhanced computed tomography image shows the abnormal relationship between the superior mesenteric artery (red circle) and the superior mesenteric vein (blue circle). (B) In a successive section in the same phase, the superior mesenteric vein (blue star) rotates around the superior mesenteric artery (red star) from posterior to anterior.

**Figure 10.**
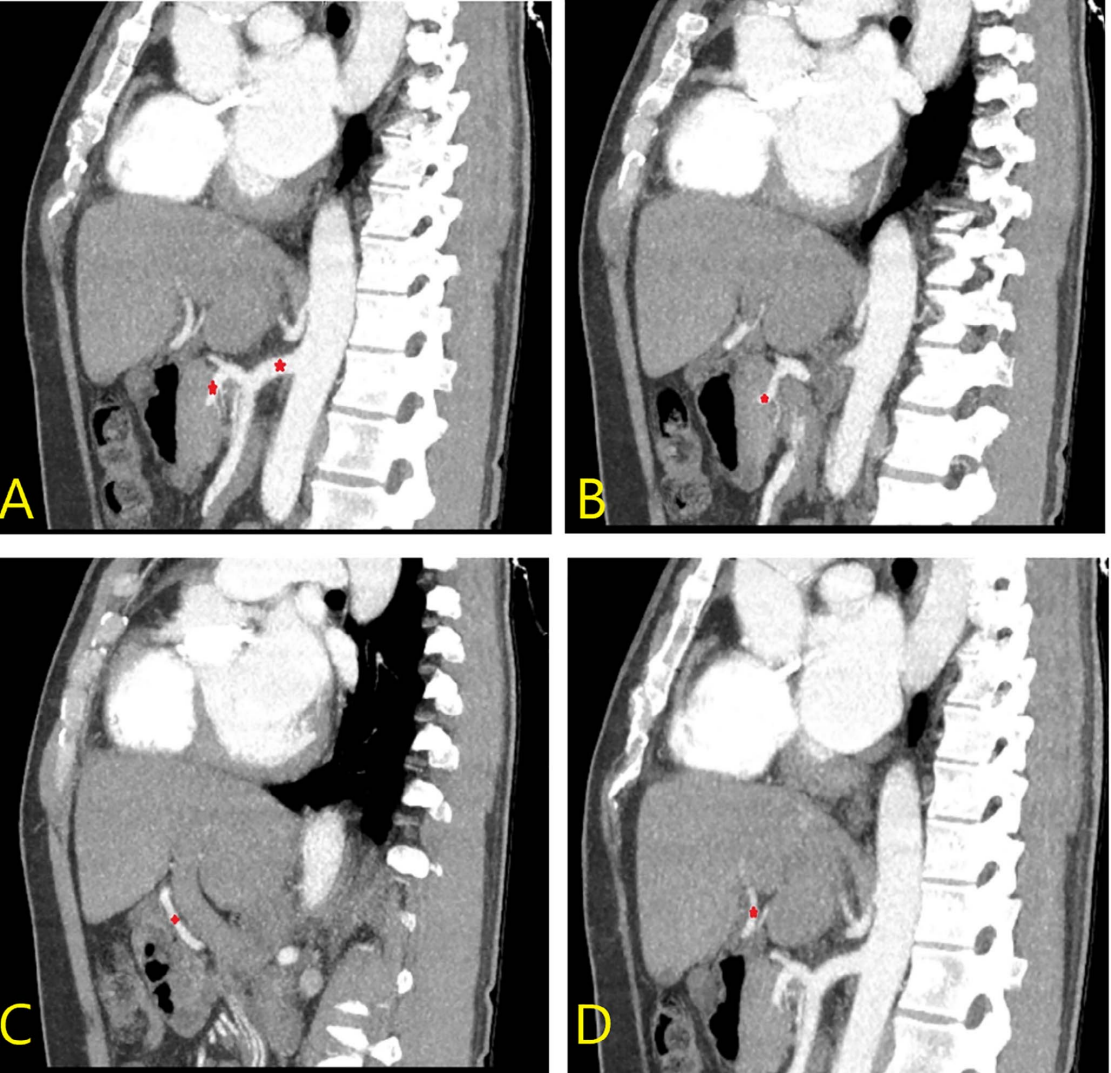
Thin-section (1 mm), sagittal multiplanar reformatted contrast-enhanced computed tomography images show in arterial phase (A) the common hepatic artery branch originating from the superior mesenteric artery, and in consecutive sections (B, C, D), the course of the common hepatic artery toward the liver, consistent with the variant hepatic arterial anatomy. In all images, the common hepatic artery is marked with a red star.

Another unusual finding was that the patient's hepatic artery branched from the superior mesenteric artery instead of the celiac trunk. The positions of the superior mesenteric artery and superior mesenteric vein were abnormal (Figures 9 and 10), with the superior mesenteric vein rotating from the posterior to the anterior of the superior mesenteric artery at the level of the L3 vertebra.

In terms of the mediastinal organ locations, the patient's heart was in the normal anatomic position on the left of the mediastinum, yet cardiomegaly was observed. The lungs appeared normal on contrast-enhanced thorax CT. The aorta, pulmonary arteries, and pulmonary venous vasculature were in their natural anatomic positions. Duplication of atria or bilobed lung, frequently observed in heterotaxy syndrome, was not observed in this patient. Transthoracic echocardiography revealed a functional mechanical aortic valve, mild paravalvular aortic regurgitation, moderate eccentric mitral insufficiency, left atrium dilatation (56 mm), and left ventricular hypertrophy. The right ventricle and right atrium were dilated. Ejection fraction was normal.

The genitourinary system appeared normal, as in most patients with heterotaxy syndrome. Neither heterotaxy syndrome nor any similar medical history was present in the patient's family, nor was there any history of consanguineous marriage.

The patient had no history of chronic cough, nasal congestion, or frequent respiratory tract infections that can be observed in patients with ciliary anomalies associated with heterotaxy syndrome. The patient had no history of recurrent pneumonia, suggestive of functional hyposplenia, and no chest pain indicative of pulmonary embolism resulting from deep vein thrombosis. Both clinical problems can be seen in patients with heterotaxy syndrome. Although subileus of the colon was observed on imaging, the patient's abdominal examination was normal with no symptoms such as abdominal pain or constipation.

Following general care guidelines,^5^ the patient was referred for genetic counseling. Because the patient did not have hyposplenia, he was not referred to follow-up with an immunologist. During the patient's follow-up, liver function tests remained at similar levels. Because no increase was observed, additional medication and biopsy were not planned. Because the patient's low-density lipoprotein level was >200 mg/dL, continued use of atorvastatin and an appropriate diet were recommended.

## DISCUSSION

The formation of the visceral organs occurs during the early mesoderm development period. Growth signal molecules must be directed adequately and accurately for normal development. In patients with heterotaxy syndrome, a disruption of organ development early in the growth of the embryo typically causes right or left isomerism of the viscera, resulting in duplication of one side and malrotation of the thoracic and abdominal organs. Any disorder occurring in this embryologic growth process may cause heterotaxy abnormality. Although more than 80 genes associated with organ development have been identified,^6^ the occurrence of genetic anomalies remains unknown.^1^

An important feature to evaluate in heterotaxy is isomerism, which means mirrored organs, that can be defined as mirrored embryonic atrial appendages (such as bilateral morphologic right atrial appendages in right isomerism).^7^ Patients with left isomerism have bilateral left-sidedness with polysplenia, bilobed lungs, and a right-sided stomach. The continuity of the inferior vena cava with the azygos vein is common.

On the other hand, patients with right isomerism are usually asplenic and have bilateral right atria and trilobed lungs. Right isomerism is generally accompanied by pulmonary venous anomalies. A duplication of either the right or left results in a misalignment between the pulmonary venous vasculature, as well as ventricles that are morphologically discordant with the atrium.

Right isomerism is more common in males, while left isomerism is more common in females.^6^ The Table lists different organ system abnormalities observed in patients with heterotaxy syndrome.

**Table.** Organ System Anomalies in Patients With Heterotaxy Syndrome^2^

Cardiovascular System	Gastrointestinal System	Respiratory System	Central Nervous System	Genitourinary System	Immune System
Position of the heart: dextrocardia/mesocardia Interrupted inferior vena cava Bilateral right/left atrium Atrioventricular septal defects Abnormal pulmonary circulation Right/left outflow tract anomalies Abnormalities in hepatic veins/extrahepatic portocaval junctions	Intestinal malrotation Biliary atresia Midline/left liver Polysplenia/asplenia Duodenal atresia Annular pancreas/agnesis of dorsal pancreas	Bilateral bilobed/trilobed lungs Bronchiectasis Sinopulmonary infections	Hydrocephalus Absence of corpus callosum Holoprosencephaly Meningomyelocele	Horseshoe kidney Dysplastic/hypoplastic kidney	Immune deficiency

### Clinical Findings

Because cardiac anomalies in heterotaxy syndrome can be severe, heterotaxy is generally diagnosed early in life, often during the neonatal period.

Buca et al compiled the prevalence of anomalies in 647 fetuses with a prenatal diagnosis of isomerism from 16 published studies.^8^ The cardiovascular system was the most commonly malformed system, with atrial septal defect being the most frequently observed in both left isomerism (pooled proportion [PP] 59.3%, 95% CI 44.0%-73.7%) and in right isomerism (PP 72.9%, 95% CI 60.4%-83.7%). Buca et al observed ventricular septal defect in 27 of 233 cases of fetuses with left isomerism syndrome and detected atrial septal defect in 22 of 233 fetuses.^8^ The prevalences of stomach and liver malposition were 59.4% and 32.5%, respectively, in fetuses with left isomerism, and 54.5% and 45.9%, respectively, in fetuses with right isomerism.^8^

In a 1983 review by Peoples et al of 146 autopsies of patients with polysplenia syndrome, at least 50% of the patients had cardiac anomalies, and 75% died before they reached 5 years of age.^9^ A 2020 study showed that the long-term prognosis for heterotaxy syndrome is still poor; the survival rate has not dramatically improved even with modern surgical interventions (the overall mortality was 40.2% in a cohort of 264 patients), and risk factors associated with the syndrome have not decreased.^10^

Gottschalk et al retrospectively reviewed information on 165 fetuses diagnosed with heterotaxy during the prenatal period.^11^ Left isomerism was present in 112 cases (67.9%) and right isomerism in 53 cases (32.1%). Ninety-seven of the 112 cases with left isomerism had a cardiac anomaly (86.6%), and all 53 cases with right isomerism had a cardiac defect (100%). This finding implies that patients with right isomerism have a high risk for cardiac defects and thus an increased risk of mortality. In the Gottschalk et al study, atrioventricular septal defect was the most common cardiac anomaly in both groups: 71.4% in those with left isomerism and 67.9% in those with right isomerism. Gottschalk et al did not report an incidence for atrial septal defect.^11^

Our patient's heart was located in the normal anatomic position on the left side. His cardiomegaly was ascribed to a history of aortic insufficiency. To our knowledge, ours is the first case to report heterotaxy syndrome with sinus venosus type atrial septal defect. Although the patient's right atrium and right ventricle were enlarged, he had no embryologic developmental anomalies. Because the patient had a bicuspid aortic valve that developed into aortic valve insufficiency (stages C and D) and ascending aortic aneurysm, he underwent valve replacement and aortic tube graft surgery at age 31 years. However, he had no other large vessel anomaly commonly seen in polysplenia syndrome cases. His pulmonary circulation and anatomic structures were normal. Consequently, the patient went undiagnosed for decades.

**Figure 11.**
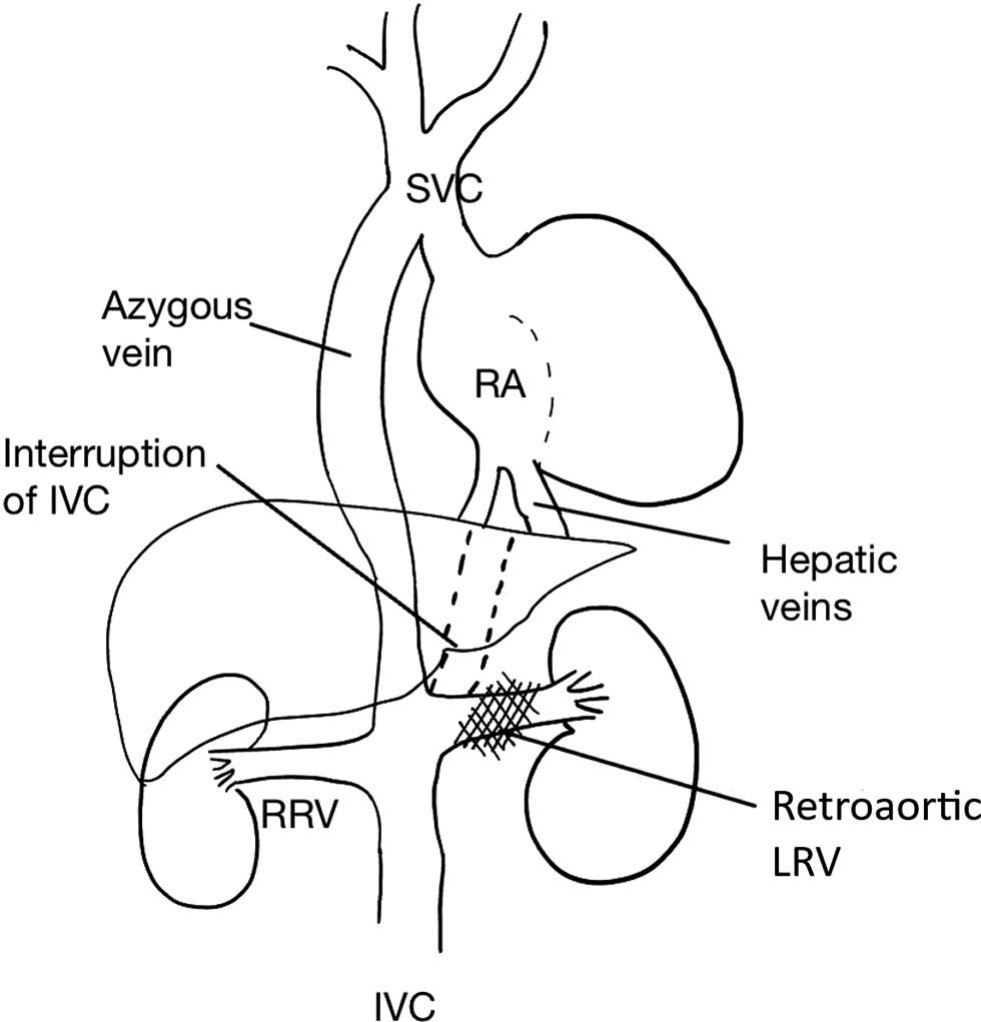
**Schematic representation of vena cava anomaly.** IVC, inferior vena cava; LRV, left renal vein; RA, right atrium; RRV, right renal vein; SVC, superior vena cava.

Inferior vena cava anomalies are common in patients with heterotaxy syndrome. Left inferior vena cava, double inferior vena cava, circumaortic left renal vein, interruption of the inferior vena cava, continuity with the azygos or hemiazygos vein, absent infrarenal inferior vena cava, and circumcaval ureter are anomalies seen in cases of heterotaxy polysplenia syndrome.^12^ Buca et al detected interruption of the inferior vena cava in the majority of polysplenia cases (PP 89.2%, 95% CI 80.5%-95.5%).^8^ In our patient, the inferior vena cava was interrupted in the infrarenal region and continued as the azygos vein (Figure 11). An interrupted inferior vena cava is a rare congenital anomaly, and the azygos continuation of the vena cava has a prevalence of 0.2% to 3% in the general population.^13^

In our patient, the renal vein was retroaortic, a vascular variation unrelated to heterotaxy.^14^ The position and size of both kidneys were normal. His hepatic veins drained directly into the right atrium.

In cases of polysplenia syndrome, multiple spleens are seen in the abdomen.^6^ In our patient, 8 spleens were widely distributed in the right upper quadrant of the abdomen. Buca et al reported the incidences of stomach and liver malposition in fetuses with left isomerism to be 59.4% (95% CI 38.1%-79.0%) and 32.5% (95% CI 11.9%-57.6%), respectively.^8^ In the study by Burton et al, 58.1% of patients diagnosed with heterotaxy syndrome had the liver symmetrically positioned in the midline of the body.^15^ In our patient, the stomach was on the right, opposite of normal anatomy, and the liver was located in the midline, almost completely filling the right and left upper quadrants because of its size. Our patient's biliary system was normal.

In the Buca et al study,^8^ only 14.2% (95% CI 2.5%-33.1%) of the patients with left isomerism had intestinal malrotation. However, Burton et al observed intestinal malrotation in 60.4% of 116 autopsy cases.^15^ Our patient had intestinal malrotation. His duodenojejunal junction was posteroinferior to the duodenal bulb, the jejunal loops were in the lower left quadrant, and the ileal loops were in the upper left quadrant. There was no volvulus, but our patient had asymptomatic subileus.

Similar to the spleen, the pancreas develops from the dorsal mesoderm; therefore, abnormalities in both organs can be detected together in heterotaxy. Our patient had an annular pancreas.

### Follow-Up and Treatment

Because multiple organ systems can be malformed in heterotaxy syndrome, there is no single approach to patient management. Systemic involvement can include cardiac, immunologic, gastrointestinal, genitourinary, respiratory, venous, and central nervous systems. Treatment and surgical intervention decisions should be based on a multidisciplinary approach and the patient's unique findings.^16^

The heart is the most important asymmetric organ associated with complications.^8,9,11^ Congenital heart defects associated with heterotaxy syndrome include atrial septal defects, total anomalous pulmonary venous connection, and inferior vena cava interruption with azygous or hemiazygos continuation.^8,9,11,12^ Common indications for surgical intervention in infants are total anomalous pulmonary venous return and severe cyanosis, but Khan et al reported a discharge mortality of 38% in infants who underwent total anomalous pulmonary venous connection repair.^17^ Patient survival is largely dependent on the severity and nature of the cardiac anomaly; consultation with a cardiologist and cardiac surgeon is necessary.

The malformed immunologic system is another clinically important system because patients with heterotaxy are at high risk for bacteremia, regardless of anatomic splenic type.^16,18^ Piano Mortari et al reported that the rate of hospitalization for infections was 50% in asplenic patients compared to 8% in patients with a normal spleen or polysplenia.^19^ In a study of 29 pediatric patients with heterotaxy, 24% of the patients had a total of 8 sepsis events, 86% of those patients developed sepsis even with antibiotic prophylaxis, and the overall mortality rate was 44%.^20^ Another study of 95 patients with heterotaxy showed that these patients were at high risk for community-acquired severe bacterial infection and had a mortality rate of 31%.^21^ Thus, determining splenic functionality is crucial. Two methods of determining splenic function are detection of Howell-Jolly bodies by light microscopy and technetium-99m scintigraphy. Asplenic individuals are prone to infections, particularly infections caused by encapsulated bacteria such as *Streptococcus pneumoniae, Haemophilus influenzae*, and *Neisseria meningitidis*. Consequently, people with asplenia are frequently advised to take preventive measures, including vaccinations and prophylactic antibiotics, to lower their risk of such infections. In individuals with polysplenia, splenic functionality is variable. Dysfunction must be evaluated individually, and if necessary, the patient should be referred to an immunologist. Vaccination and boosters for encapsulated bacteria are strongly recommended for individuals with hyposplenia, and the need for prophylactic antibiotic use should be determined by an infection specialist.

Another commonly malformed organ system in heterotaxy syndrome is the gastrointestinal system. Associated risks are volvulus and compression of the vascular compartment. Because of the high incidence of malrotation in heterotaxy syndrome, some institutions routinely screen asymptomatic patients.^22^ Symptomatic patients require surgical intervention (Ladd procedure).^22^ However, the approach for asymptomatic patients is controversial; postponing surgery in asymptomatic patients may increase mortality, but Elder et al reported a high mortality risk of the operation (13%) attributable to cardiovascular complications.^23^ Landisch et al recommend that a Ladd procedure should only be performed on patients with heterotaxy syndrome who have the greatest risk of volvulus.^22^ Prophylactic Ladd procedure is not recommended because of the mortality risk. Our patient had a subileus, and we explained the risk of developing acute intestinal pathologies, such as the sudden presentation of volvulus or bowel obstruction, to our patient and recorded the information in his medical record for future follow-up. Physicians caring for patients with heterotaxy syndrome should be aware of the risks for the formation of acute bowel obstructions so they can provide prompt management.

Many other abnormalities, such as biliary atresia, respiratory ciliary dysfunction, and neuropsychiatric findings, can be detected in patients with heterotaxy.^16^ Thus, all organ systems and vascular structures should be evaluated separately and monitored according to the patient's condition.

## CONCLUSION

As shown in our case, the diagnosis of heterotaxy syndrome can be delayed until adulthood. Although heterotaxy syndrome is usually diagnosed at an early age, the absence of severe clinical findings can delay diagnosis. This case contributes to the limited literature on this rare abnormality. The treatment and follow-up of patients with heterotaxy syndrome are not standardized and must be personalized according to each patient's unique symptoms and findings.

## Notes

*This article meets the Accreditation Council for Graduate Medical Education and the American Board of Medical Specialties Maintenance of Certification competencies for Patient Care and Medical Knowledge.*
